# Larvicidal and Biochemical Effects of *Ipomoea carnea* (Convolvulaceae) Methanolic Leaf Extract Against *Spodoptera frugiperda* (Lepidoptera: Noctuidae) Under Laboratory Conditions

**DOI:** 10.3390/insects17090888

**Published:** 2026-08-25

**Authors:** Eman K. Amin, Talaat E. Emara, Gamal M. Hassan, Tahany M. Eid, Hanem H. Sakr

**Affiliations:** 1 Department of Zoology, Faculty of Science, Menoufia University, Shebin El-LKom 32512, Menoufia, Egypt; aboushanab@science.menofia.edu.eg (E.K.A.); prof.talaatemara2026@gmail.com (T.E.E.); tahanyeid@gmail.com (T.M.E.); 2 Plant Protection Research Institute (PPRI), Agricultural Research Center (ARC), Dokki, Giza 12611, Egypt; dr.jimy.hassan@gmail.com

**Keywords:** *Ipomoea carnea*, *Spodoptera frugiperda*, larvicidal activity, biochemical analysis

## Abstract

The larvicidal and biochemical effects of *Ipomoea carnea* Jacq. (Convolvulaceae) methanol leaf extract against the fall armyworm, *Spodoptera frugiperda* (Lepidoptera: Noctuidae) were determined under standard laboratory conditions. The second and fourth instar larvae of *S. frugiperda* were fed on castor bean leaves (*Ricinus communis*) treated with different concentrations (1–5%) of *I. carnea* methanolic leaf extract. The mortality percentage significantly increased in correlation with both concentration and exposure time compared to control. Second instar larva of *S. frugiperda* was more susceptible to this extract than the fourth instar, with LC_50_ values of 0.968% and 1.052%, respectively. Castor bean leaves treated with 5% extract and fed to second and fourth larval instar caused 96.3 and 92.9% larval mortality, respectively. Results from the biochemical analysis of larvae treated with *I. carnea* methanolic leaf extract revealed a significant reduction in total content of carbohydrate, protein and lipid compared to control. *I. carnea* methanolic leaf extract induced significant inhibition of acetylcholinesterase, α-esterase and β-esterase activities, alongside increase in peroxidase activity compared to the control groups. The current results are consistent with neurophysiological disruption and activation of oxidative stress in the treated *S. frugiperda* larvae. Therefore, we recommend *I. carnea* methanolic leaf extract for controlling *S. frugiperda*. Further field investigation into the efficacy of *I. carnea* methanolic leaf extract against *S. frugiperda* and the non-target organisms was required.

## Simple Summary

The fall armyworm, *Spodoptera frugiperda*, is one of the most destructive insect pests affecting maize and several other crops worldwide. Excessive use of synthetic insecticides has led to environmental concerns and the development of insecticidal resistance. Therefore, there is a dire need for environmentally friendly alternatives. *Ipomoea carnea* methanolic leaf extract was evaluated (under standard laboratory conditions) against the second and fourth instar larvae of *S. frugiperda*. The extract caused high larval mortality, reduced larval and pupal growth and induced developmental abnormalities. The extract decreased AChE and esterase activity and increased peroxidase activity. These findings are consistent with neurophysiological disruption and activation of oxidative stress. Chemical analyses of *Ipomoea* using HPLC and GC–MS revealed the presence of several bioactive compounds that may contribute to the observed insecticidal activities. These findings suggest that *I. carnea* methanolic leaf extract could be promising convenient insecticide against *S. frugiperda*. Further field investigation into the efficacy of *I. carnea* methanolic leaf extract against *S. frugiperda* and the non-target organisms was required.

## 1. Introduction

*Spodoptera frugiperda* (J. E. Smith, 1797) (Lepidoptera: Noctuidae), is a highly invasive and destructive lepidopteran pest worldwide. Native to the Americas, *S. frugiperda* has spread rapidly to Africa, Asia and more recently to Europe. It has successfully established itself across Sub-Saharan Africa where it continues to disrupt agriculture, particularly smallholder cereal production [1]. In Egypt, this pest spread rapidly across various governorates [2,3] following its first detection in June 2019 from Kom Ombo and Edfu fields in Aswan governorate [4,5]. Usually, the early instar larvae of *S. frugiperda* infest the lower side of the host’s leaves, feeding on them and leaving distinctive holes patterns. The late larval instars of this pest eat the whole leaves of the host except the blade midribs. A two-season comparative field study evaluating the effect of Emamectin benzoate on *S. frugiperda* infested maize, *Zea mays* (L) was conducted by [6] at Nubaria, Behaira Governorate, Egypt. They stated that a maize field treated with Emamectin benzoate yielded 4.249 and 3.416 T/Fed at the first and second year, respectively. However, the untreated maize field yielded 3.30 and 2.69 T/Fed, respectively [6].

Maize, *Zea mays* (L), is the most suitable host plant with heavy damage and high yield losses, especially in severe infestation by *S. frugiperda,* which reached 70–73% under uncontrolled field conditions [1,7]. In addition to maize, the larvae also attack a wide range of other economically important crops, including sorghum, wheat, peanut, soybean, cotton, and fodder grasses in some regions [8,9,10]. The use of chemical insecticides remains a common approach for controlling *S. frugiperda* for their rapid effectiveness in suppressing pest populations and minimizing crop losses. Nevertheless, heavy dependence on these chemicals poses serious ecological and agronomical challenges. In its native range in the Americas, intensive chemical control of *S. frugiperda* led to the development of resistance to numerous commercial insecticides before its arrival in Africa [1]. These negative impacts also include environmental contamination, and deleterious impacts on beneficial organisms. Such reliance ultimately disrupts ecosystem stability and threatens the long-term sustainability of integrated pest management strategies [11]. Biological control agents have been widely explored as sustainable, cost-effective and eco-friendly alternatives. These alternatives utilize natural enemies, bio-pesticides and plant extracts to suppress pest populations [12]. Many scientists are researching bio-insecticide based botanical extracts that have short residual effects, are less persistent in the soil environment, have less selectivity and originate from sustainable raw materials [13].

The pink morning glory, *Ipomoea carnea* Jacq. (Convolvulaceae) is a perennial shrub native to South America, but it is widely distributed across tropical and subtropical regions globally. The aqueous extract of *I. carnea* is a promising source of bioactive compounds such as alkaloids, flavonoids, tannins, saponins, phenols and terpenoids with insecticidal activity against *Aedes aegypti* [14]; *Aphis craccvora*, *Bemisia tabacci* [15]; *Spodoptera littoralis* [16] and *S. litura* [17]. The methanolic leaf and flower extracts of *I. murucoides* caused mortality in neonate *S. frugiperda* larvae, prolonged development and reduced both larval weight and female fecundity [18].

The current study aims to evaluate the larvicidal activity of *I. carnea* methanolic leaf extract against the second and fourth instar larvae of *S. frugiperda* under laboratory conditions. Furthermore, the study investigated the biochemical and enzymatic alterations induced in treated larvae.

## 2. Materials and Methods

### 2.1. Plant Sample Collection and Extraction

Fresh leaves of *Ipomoea carnea* L. (Convolvulaceae) were collected from a field in Saft El-Laban, Giza, Egypt (30.0288° N latitude and 31.1702° E longitude) in March 2023. The plant sample was carefully rinsed in tap water (to eliminate dust and surface contaminants) and air-dried in the shade at room temperature until most of the leaves’ moisture evaporated, without allowing the leaves to become completely dry. For leaf extraction: 500 g of leaves were subsequently homogenized using an electric blender to form a coarse paste. This paste was soaked in 1.5 L of absolute methanol in a glass conical flask for seven days (at room temperature) with occasional shaking. Plant extract was filtered through Whatman filter paper No. 1, and the filtrate was concentrated under reduced pressure using a rotary evaporator at 45 °C. The crude extract was further evaporated to yield crude methanolic residue, which was stored at −10 °C until use. A 5% (*w*/*v*) stock solution of crude methanolic extract was prepared using absolute methanol as a solvent.

### 2.2. Phytochemical Analysis of I. carnea Methanolic Leaf Extract Using HPLC and GC–MS

The phytochemical profile of *I. carnea* methanolic leaf extract was investigated using both high-performance liquid chromatography (HPLC) and gas chromatography–mass spectrometry (GC–MS) techniques (Egyptian Atomic Energy Authority, Chemistry Research Department). Phenolic acids and flavonoid compounds were analyzed using a Vanquish™ Core HPLC system (a Thermo Scientific™ Vanquish™ Core HPLC system (Thermo Fisher Scientific Inc., Waltham, MA, USA), equipped with an autosampler, solvent degasser, UV/Vis detector and Chromeleon™ Chromatography Data System (CDS).

Chromatographic separation was performed on a reversed-phase C18 column (125 mm × 4.60 mm, 5 µm particle size). Detection wavelengths were set at 250 nm for phenolic acids and 360 nm for flavonoids. Phenolic acids were separated using a gradient mobile phase consisting of methanol (solvent A) and acetic acid in water (1:25, *v*/*v*) (solvent B). The gradient program started with 100% solvent B for 3 min, followed by 50% solvent A for 5 min. Solvent A was then increased to 80% for 2 min and subsequently returned to 50% for an additional 5 min. Flavonoids were analyzed using an isocratic elution system with acetonitrile (A) and 0.2% (*v*/*v*) aqueous formic acid (B) at a ratio of 70:30 (*v*/*v*). The flow rate was maintained at 1 mL/min, the column temperature at 25 °C and the injection volume was 25 µL.

The chemical composition of the methanolic leaf extract of *I. carnea* was further analyzed using a GC–MS system (GC-TSQ mass spectrometer, Thermo Scientific, Austin, TX, USA). Separation was carried out on a TG–5MS capillary column (30 m × 0.25 mm internal diameter, 0.25 µm film thickness). The oven temperature program was initially set at 60 °C, then increased at a rate of 5 °C/min to 250 °C and held for 2 min, followed by a further increase to 300 °C at 30 °C/min. The injector temperature was maintained at 270 °C. Helium was used as the carrier gas at a constant flow rate of 1 mL/min. A diluted sample (1 µL) was injected automatically using an AS3000 autosampler (Thermo Fisher Scientific Inc., Waltham, MA, USA) in split mode, with a solvent delay of 4 min. Mass spectra were obtained using electron ionization (EI) at 70 eV over a mass range of m/z 50–650 in full scan mode. The ion source and transfer line temperatures were set at 200 °C and 280 °C, respectively. Compound identification was achieved by comparing the obtained mass spectra with those available in the WILEY 09 and NIST 14 mass spectral libraries.

### 2.3. Rearing of Spodoptera frugiperda

A laboratory strain of *Spodoptera frugiperda* (Lepidoptera: Noctuidae) was kindly provided by Prof. D./Hassan G. M. (Plant Protection Research Institute, Dokki, Egypt). The insect culture was established in the Insect Laboratory, Department of Zoology, Faculty of Science, Menoufia University, Egypt. *S. frugiperda* larvae were kept in plastic containers measuring 20 × 10 × 7 cm, each of which was lined with a fine layer of sawdust to control moisture and covered with muslin tied around the neck with rubber bands. A fresh leaf of *Ricinus communis* (Euphorbaceae) with a green midrib blade was used for larval feeding. These containers were maintained at controlled conditions of temperature and humidity (25 ± 2 °C and 65 ± 5% R.H.) until pupation.

The insect pupae were placed in glass jars (20 cm in height × 8 cm in diameter) containing a thin layer of fine sawdust and covered with muslin tied around the neck with rubber bands and kept at the same environmental conditions mentioned above until adult emergence. The newly emerged adults were supplied with a 50% honey solution absorbed on a cotton pad as a food source. Each jar was fitted with a zigzag-folded paper sheet to serve as an ovipositional site, which was replaced daily. The collected egg masses were then transferred to plastic containers and kept until hatching. After six consecutive generations, a laboratory insect colony was acquired, which is suitable for the current bioassay experiments.

### 2.4. Toxicity of Ipomoea carnea Methanolic Leaf Extract Against S. frugiperda Larvae

The leaf-dipping method was employed (under laboratory conditions) to ascertain the insecticidal activity of *I. carnea* methanolic leaf extract against the second and fourth larval instars of *S. frugiperda*. The first instar was excluded due to its extreme sensitivity, which could result in excessive mortality and unreliable data. The third instar was excluded as it is not representative of the mid-late larval stage. The second and fourth instar larvae of *S. frugiperda* were selected to represent early and mid-late larval stages for evaluating stage-dependent susceptibility to the methanolic extract of *I. carnea*. The second instar larva was further used for LC_50_-based post-treatment observations to evaluate the latent toxic developmental effects throughout the subsequent larval development up to the F1 adult.

Three replicates were conducted under laboratory conditions, each of which consisted of 20 larvae. Five serial concentrations (*w*/*v*) of the extract (1%, 2%, 3%, 4% and 5%) were prepared in methanol. For each replicate, twenty fresh castor bean leaves, *Ricinus communis* L. (4 × 4 cm), were immersed in each concentration for 30 s and air-dried. For control groups, the leaves were dipped in distilled methanol only. Each larva was allowed to feed on treated leaves for 24 h in a glass petri dish (10 cm diam.). Both treated and control larvae were individually transferred into plastic boxes (7.5 × 7.5 × 4 cm), maintained under the controlled laboratory conditions mentioned above and fed fresh leaves until pupation. The number of live and dead larvae in each group was recorded after 6, 12, 24, 36, 48 and 72 h post-treatment. Live larvae in each treated and control group were weighed individually after 24 h of every instar. The percentage of larval mortality, pupation, adult emergence and malformations were calculated. Larval mortality data was corrected relative to mortality in control using Abbot’s formula [19]. Reduction in larval and pupal weight was calculated according to [20].

Weight reduction (%) = [(Mean weight of control − Mean weight of treated)/Mean weight of control] × 100

### 2.5. Effect of I. carnea Methanolic Leaf Extract on Some Biochemical Components of S. frugiperda Larvae

The effects of *I. carnea* methanolic leaf extract on some biochemical components of *S. frugiperda* larvae were determined. For sample preparation, after 24 h of treatment the whole larval body of fourth instar *S. frugiperda* larvae were homogenized and prepared for biochemical assays. Samples were immediately frozen and stored at −20 °C until analysis. No protease inhibitors were used during sample preparation. The biochemical analyses of *S. frugiperda* treated and control groups were conducted at the Insect Physiology Department, Plant Protection Research Institute, Agricultural Research Center, Giza, Egypt. Total protein content was determined according to [21], while total lipids were estimated following the method of [22]. Total carbohydrates were extracted and quantified using methods described by [23] and expressed as mg/g body weight. The activity of acetylcholinesterase (AChE) was determined using the method of [24] and the results were expressed as nmol of substrate hydrolyzed/min/mg of protein. The activities of α- and β-esterases were determined following the procedure of [25], using α-naphthyl acetate and β-naphthyl acetate as substrate compounds, and expressed as nmol/min/mg protein. Esterases are key enzymes involved in the metabolism and detoxification of xenobiotic compounds, including plant-derived secondary metabolites. Peroxidase activity was determined according to [26]. Three replicates of five larvae were used in each treatment.

### 2.6. Statistical Analysis

All data were expressed as mean ± standard error (SE). The lethal concentration values (LC_50_, LC_90_, LC_95_ and LC_99_), toxicity index and resistance ratio were calculated using the Ldp-line software (http://www.ehabsoft.com/ldpline, access on 25 February 2024) computer program [27] based on probit analysis according to [28]. The significant differences between means (each mean consisted of three replicates, each replica contained 20 larvae in randomized complete block design) among treatments at each time interval (6, 12, 24, 36, 48, 72 h) were determined using one-way analysis of variance (ANOVA). The *t*-test analysis was used to determine the variance between weight gained in the larval (full developed) and pupal stages of the treated and control groups. Differences were considered statistically significant at *p* < 0.05.

## 3. Results

### 3.1. Toxicity of Ipomoea carnea Methanolic Leaf Extract Against Spodoptera frugiperda Larvae

The potential of *Ipomoea carnea* methanolic extract against second instar larvae of *Spodoptera frugiperda* was ascertained under laboratory conditions. The results showed that the percentage of *S. frugiperda* larval mortality was significantly increased with both concentration and exposure time (Table 1). At a concentration of 1%, the mean mortality percentages were 30.00 ± 5.00% and 64.81 ± 10.19% after 6 h and 72 h post-treatment, respectively. The highest concentration of *I. carnea* extract (5%) elicited 71.67 and 96.30% larval mortality after 6 h and 48 h post-treatment, respectively. In contrast, the control group exhibited mortality (≤3.33%) throughout the experimental period (Table 1).

The fourth instar larvae of *S. frugiperda* treated with different concentrations of *I. carnea* methanol extract significantly (*p* ≤ 0.0001) suffered from the treatment in a concentration and time dependent manner compared to the control group (Table 2). A concentration of 1% of *I. carnea* caused larval mortality 25.00 ± 10.00 (a) and 74.56 ± 0.44 (b) after 6 and 72 h post-treatment, respectively. The highest concentration of extract (5%) resulted in 76.67 ± 1.67 (a) and 93.33 ± 6.67 at 36 and 48 h post-treatment, respectively (Table 2).

**Figure 1:**
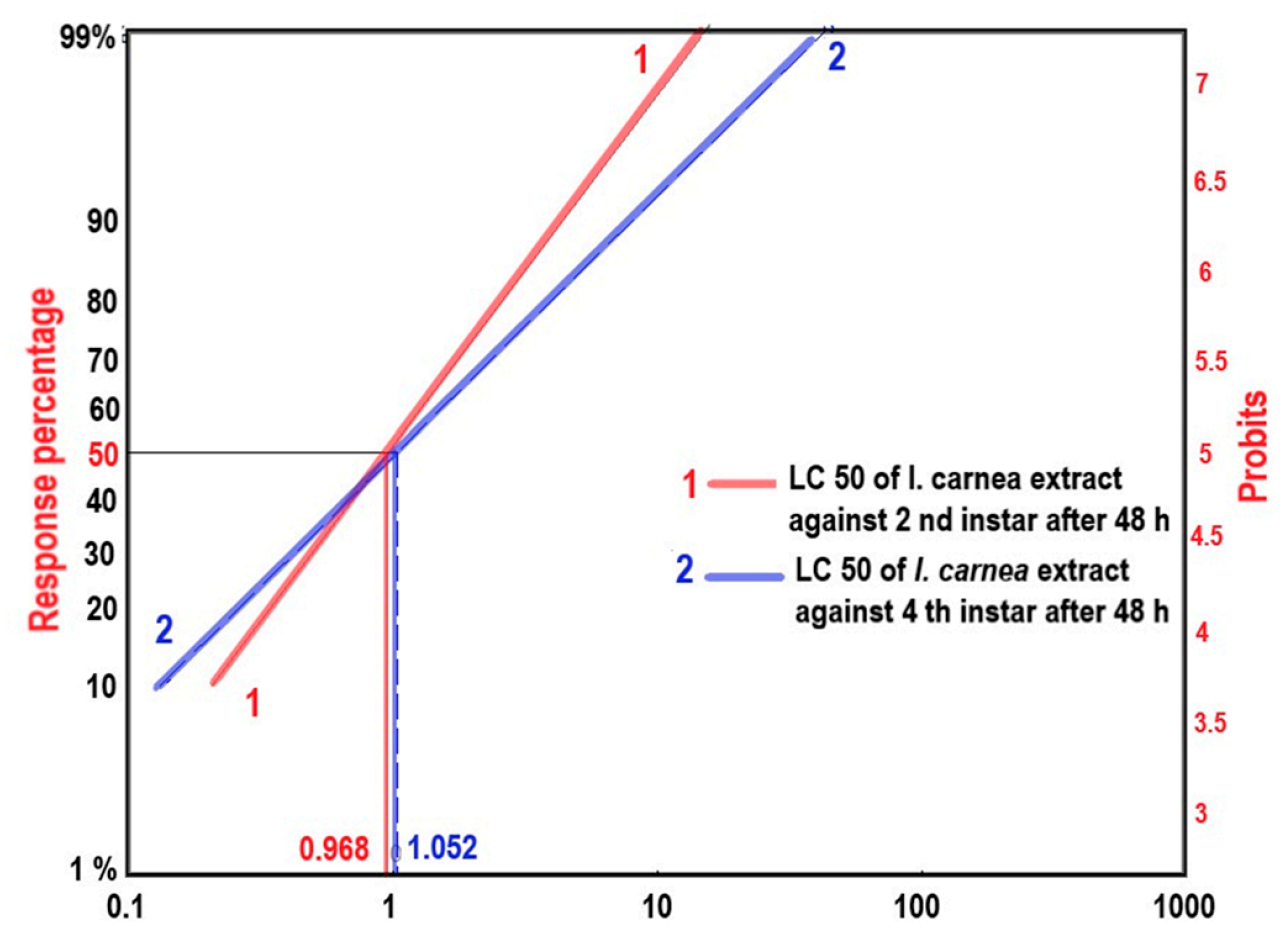
Toxicity effects of *I. carnea* methanolic leaf extract against *S. frugiperda* larvae.

After 48 h of treatment, the toxicity parameters of *I. carnea* methanolic leaf extract against the second and fourth larval instars of *S. frugiperda* were illustrated in Figure 1. The lethal concentration values (LC_50_, LC_90_, LC_95_ and LC_99_) indicated that the second instar larvae were more susceptible to this extract compared to the fourth instar (Table 3). The LC_90_ and LC_95_ values were 4.453% and 6.863% for the second instar, respectively, while it increased to 8.221% and 14.726% in the fourth instar. The LC_99_ for the fourth instar (43.95%) was nearly three times that of the second instar (15.45%), confirming a reduced susceptibility in the older larvae.

The data presented in Table 4 demonstrate that the methanolic leaf extract of *I. carnea* at LC_50_ significantly reduced the larval and pupal weights of *S. frugiperda* treated as second larval instar. The reduction in larval and pupal weights was 60.78% and 58.11%, respectively.

**Figure 2:**
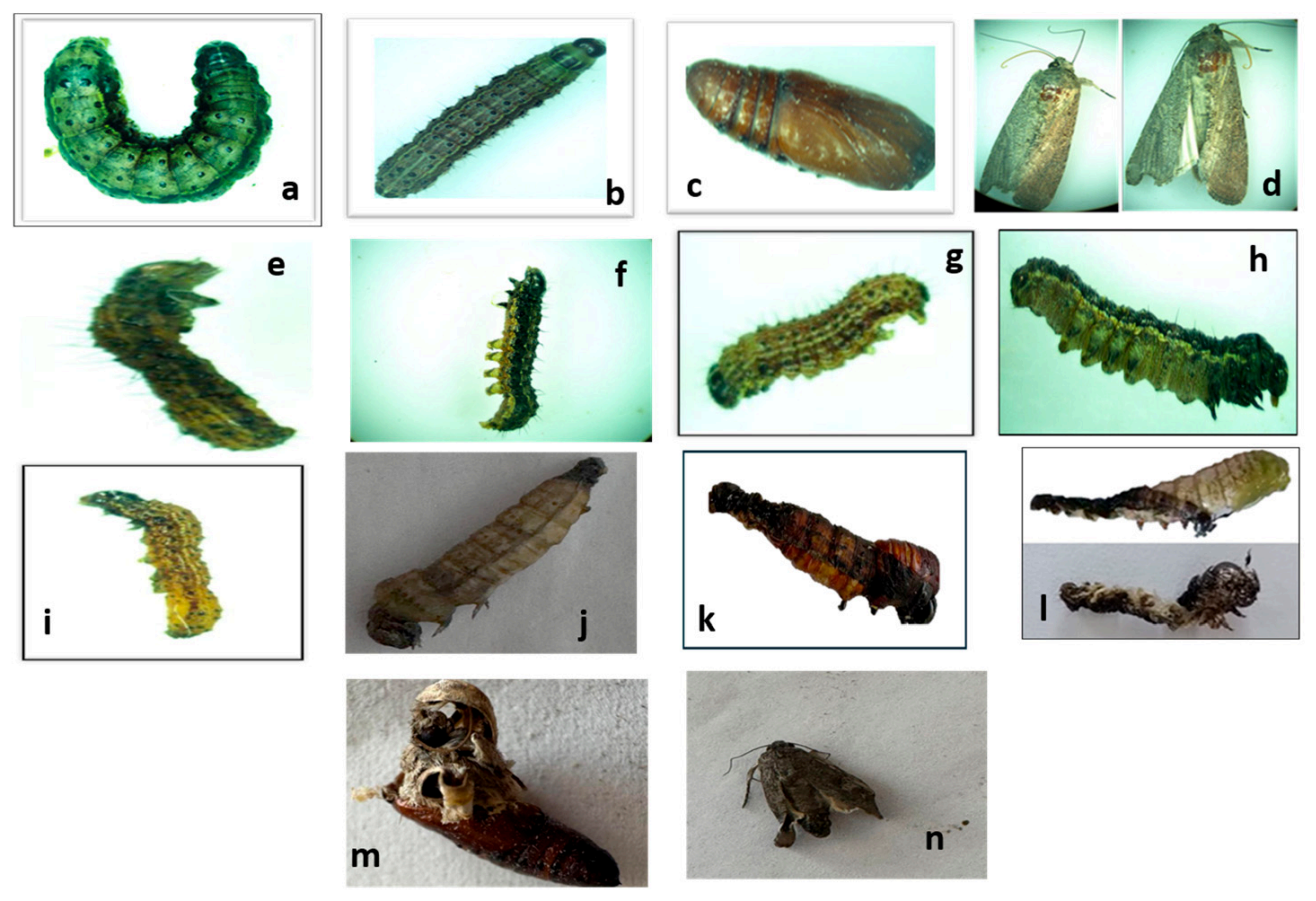
Morphological changes induced by *I. carnae* methanolic leaf extract on *S. frugiperda* larvae (**a**–**d**): healthy normal larva, pupa and adult, respectively; (**e**–**i**): abnormal treated larvae failed to moult normally and died; (**j**–**l**): abnormal treated larvae failed to moult normally and developed into an intermediate stage; (**j**–**l**): larvae–pupae intermediate; (**m**): pupa–adult intermediate; (**n**): malformed adult.

The current results indicated that the *I. carnea* methanol leaf extract markedly impaired the growth and development of *S. frugiperda* larvae. The second instar larvae treated with LC_50_ (0.968%) exhibited severe developmental disruption (Figure 2). Many individuals failed to complete moulting (ecdysis) normally and died, leading to significant reduction in the percentages of pupation and adult emergence (Table 4 and Figure 2a–n). Ten percent (*N* = 6 larvae) of treated larvae (60 larvae) failed to complete moulting normally and developed into an intermediate stage, for instance, larva–pupa intermediate (Figure 2j–l) and pupa–adult intermediate (Figure 2m). Seven percentages (*N* = 4) of treated larvae (60 larvae) developed into malformed adults (Figure 2n) compared to the untreated control groups.

### 3.2. Phytochemical Analysis of I. carnea Methanolic Leaf Extract Using HPLC and GC–MS

**Figure 3:**
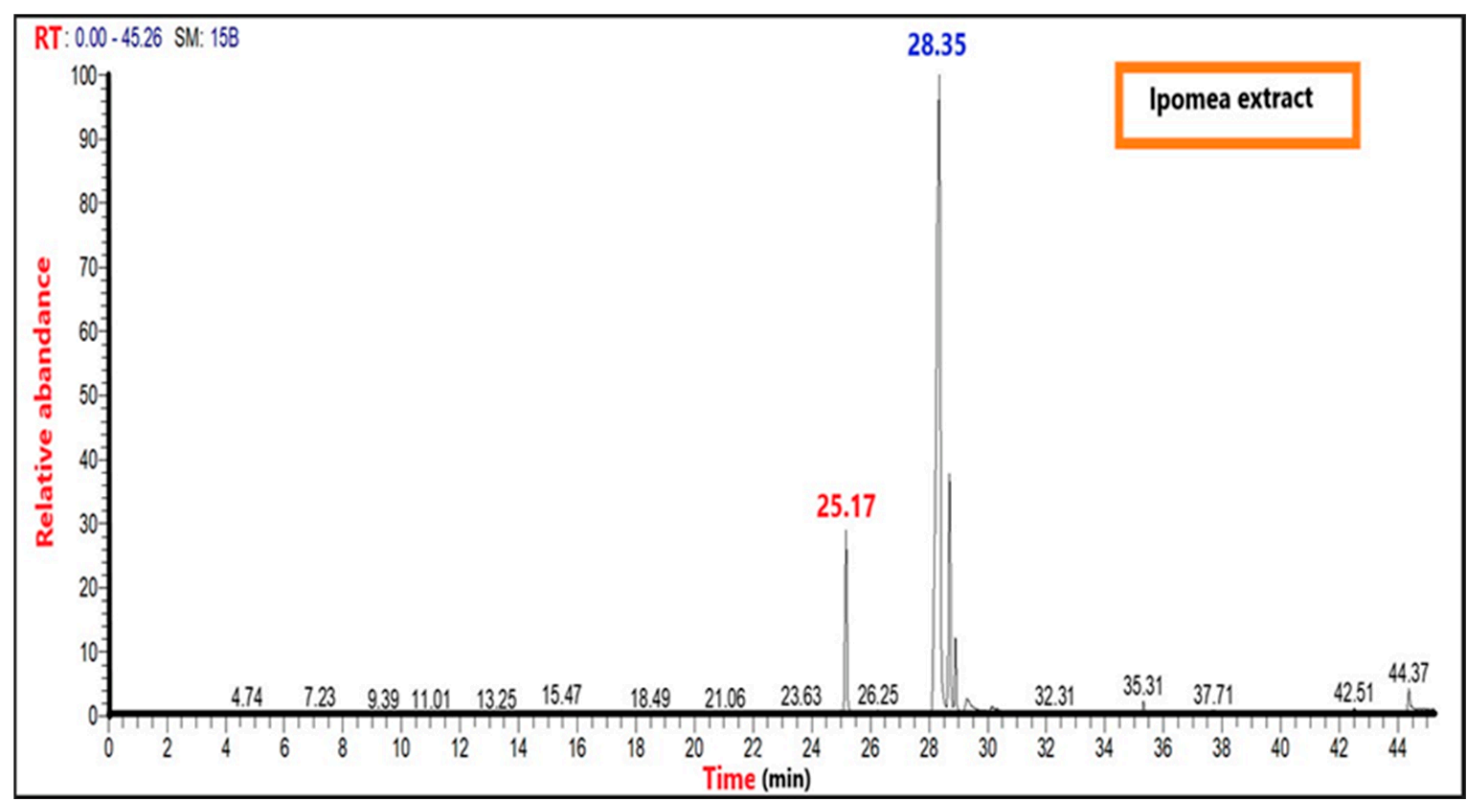
Total ion chromatogram (TIC) of *I. carnea* methanolic leaf extract obtained by GC–MS analysis, showing the separation of bioactive compounds according to their retention times.

The phytochemical profile of *I. carnea* methanolic leaf extract was investigated in the present study using both high-performance liquid chromatography (HPLC) and gas chromatography–mass spectrometry (GC–MS). These instruments ascertained that the methanol leaf extract of *I. carnea* in the current study contains many bioactive compounds (Figure 3 and Table 5). Using HPLC, seven different compounds were found (Table 5) in the extract belonging to different classes (flavonoid, flavanon glcoside, phenolic acid, hydroxycinamic acid). Table 5 showed that the extract contained ellagic acid (4.8875 µg/mL), apigenin (5.6693 µg/mL), naringin acid (11.6469 µg/mL), resveratrol (128.1935 µg/mL) and traces of transferrulic acid (0.8047 µg/mL). GC–MS analysis revealed the presence of fatty acids, ketones, aldehyde, alcohols, Pyraone, Alkene, Polycyclic sesquiterpenes, steroids, nitrogen compound (Pyrimidine-,6,diol,5-methyl-), esters, dicarboxylic acid,…etc. (Table 5).

### 3.3. Effect of I. carnea Methanolic Leaf Extract on Some Biochemical Components of S. frugiperda Larvae

The current results (Table 6) clarify the biochemical changes that occurred after treatment of fourth instar *S. frugiperda* larvae with the LC_50_ of *I. carnea* methanolic leaf extract. After 24h of treatment, a significant reduction in the total contents of carbohydrates, proteins, and lipids of the treated larvae were found. The total carbohydrate content decreased from 168.33 ± 2.03 mg/g body weight (b. wt.) to 150.67 ± 0.88 mg/g b. wt. in control and treated larvae, respectively. The total protein content markedly decreased to 343.67 ± 1.45 mg/g body weight compared to 403.67 ± 2.19 in the control group. The total lipid content showed a significant decline, from 319.33 ± 1.20 in control to 287.00 ± 1.15 mg/g b. wt. in treated larvae.

The enzyme activity of the treated larvae was affected by the LC_50_ of *I. carnea* leaf extract. The current results showed an obvious suppression of acetylcholinesterase activity from 233.33 ± 1.86 to 162.33 ± 3.28 nmol/min/mg proteins in the control and treated groups, respectively. Both α-esterase and β-esterase activities were significantly reduced in treated larvae compared to control groups (Table 6). The value of α-esterase decreased from 68.33 ± 2.03 in control to 51.00 ± 0.58 in treated larvae. The β-esterase values were 47.33 ± 1.45 and 30.67 ± 1.86 in control and treated group, respectively. In contrast, the peroxidase activity significantly increased in the treated larvae from 0.24 ± 0.01 to 0.50 ± 0.01 nmol/min/mg protein.

## 4. Discussion

### 4.1. Toxicity of Ipomoea carnea Methanolic Leaf Extract Against Spodoptera frugiperda Larvae

The present results clearly indicate strong larvicidal effects of *Ipomoea carnea* methanol leaf extract against *Spodoptera frugiperda* larvae causing larval mortality, developmental disruption and weight reduction. The larval mortality response exhibited a clear age, concentration and time-dependent pattern, suggesting the strong potential efficacy of *I. carnea* extract, particularly against the second instar larvae of *S. frugiperda*. The earlier larval instar is more susceptible to the toxic plant extract than the older ones which may be associated with differences in physiological detoxification capacity and insecticidal tolerance. These results are consistent with those of Xie et al 2025 [56]. They investigated the changes in gene expression and metabolic processes in 2nd and 3rd instar larvae of a *S. frugiperda* after consuming tobacco and maize leaves. They stated that the majority of differentially expressed genes involved in xenobiotic biodegradation and metabolism were up-regulated genes, particularly cytochrome P450s. Generally, the older larvae consumed tobacco exhibiting greater detoxification capacity than younger instars via increasing the production of metabolites through cytochrome P450 [56].

To the best of our knowledge, studies evaluating the insecticidal activity of *I. carnea* methanolic leaf extract against *S. frugiperda* are currently limited in the available research. However, the efficacy of this plant species has been investigated against closely related *Spodoptera* species [16,17]. *I. carnea* extracts poses toxic and antifeeding effects against *S. littoralis* [16] and *S. litura* [17], causing substantial larval mortality, developmental abnormalities and reduced feeding activity [16,17].

The strong larvicidal activity of *I. carnea* methanol leaf extract observed in the present study agrees with previous reports demonstrating the effectiveness of different botanical extracts against *S. frugiperda* [18,57,58,59]. The methanolic leaf and flower extracts of *I. murucoides* caused mortality of neonate *S. frugiperda* larvae, a prolonged developmental period and a reduction in both larval weight and female fecundity [18]. Several plant extracts caused considerable larval mortality and adversely affected the growth and feeding activity of *S. frugiperda* larvae [57]. The toxicity of camphor, castor and oleander extracts and chemical control (emamectin benzoate) against the third larval instar of *S. frugiperda* was evaluated under laboratory conditions by [58]. Data revealed that the oleander extract was the most toxic among the tested plant extracts, causing 86.6% larval mortality at a concentration of 20 g/L. The reference insecticide, Emamectin benzoate, caused 100% larval mortality at a concentration of 0.8 g/L [58]. A combination of *Cyperus rotundus* (Cyperaceae) and *Piper retrofractum* (Piperaceae) crude extracts was tested against *S. frugiperda* larvae by [59]. They reported that hexane extract of *P. retrofractum* (at 10.57 ppm) mixed with *C. rotundus* dichloromethane extract (at 2,504 ppm) caused up to 98% larval mortality compared to 20% mortality for each extract used alone [59]. The toxicity of tomato and pepper leaf extracts against the second instar larva of *S. frugiperda* (leaf-dipping method) was evaluated by [60]. They reported that, under Laboratory conditions, tomato leaf extract was more toxic (LC_50_ = 2676.92 ppm) than pepper leaf extract (LC_50_ = 8182.40 ppm). LC_50_ applied under the semi-field conditions revealed that tomato leaf extract was more toxic than pepper extract [60]. The cellular immune system of *S. frugiperda* was disrupted with morphological abnormalities as a response to the stress effect of the *Mirabilis jalapa* leaf nanoemulsion [61].

The current HPLC chemical profile data of *I. carnea* methanolic leaf crude extracts indicated the presence of seven compounds: ellagic acid, apigenin, naringin acid, salicylic acid (SA), anthranilic acid, transferrulic acid and resveratrol. Of the seven compounds presented, the ellagic acid, apigenin, naringin acid, resveratrol and transferrulic acid showed insecticidal activity against different insect species [62,63,64,65,66,67,68]. In a study by [62], the ellagic acid has insecticidal activity against the larvae of *Corcera cephalonica* (Lepidoptera: Pyralidae) and *Tribolium castaneum* (Coleoptera: Tenebrionidae) causing 98 and 76% larval mortality, respectively [62]. The toxic effects of apigenin (isolated from *Jatropha gossypifolia*) against *Culex quinquefasciatus* was evaluated by [63]. They found that apigenin caused reduction in the total protein level by 92% of control while the free amino acid level was increased to 104% of control [63]. The insecticidal activity of the active compound, apigenin (isolated from *Anisomeles indica*), against *Cx quinquefasciatus* was evaluated by [64]. They reported that apigenin at a concentration of 2% caused 74 and 72% egg and larval mortality, respectively. Remarkable deformities of the developmental stages were observed, with no harmful effect on the non-target organisms (*Gambusia affinis*). A docking study showed that apigenin binds and inhibits AChE activity, which resulted in larval mortality [64]. The essential oil (EO) extracted from the *Citrus paradisi* seeds revealed mortality to *Cx quinquefasciatus* larval and adult stages with LC_50_ value of 2047.40 ppm after 60 min. [65]. Using molecular docking, the EO constituent’s deacetylnomilinic acid glucoside and especially naringin caused inhibition to the AChE activity more than the reference inhibitor [65]. Insect glutathione S-transferase (GST) and carboxylesterase (CarE) are known detoxification enzymes against xenobiotic. A [66] investigated the interaction of ferulic acid (in rice) with GST or CarE genes in the brown plant hopper, *Nilaparvata lugens* (Hemiptera: Delphacidae) [66]. They stated that ferulic acid content in the tested rice verities was associated with resistance to the brown plant hopper, *N. lugens* [66]. The insecticidal activity of resveratrol against the oriental armyworm, *Mythimna separata* was studied by [67]. They reported that resveratrol at a concentration of 600 mg L- elicited 50% mortality [67]. The methanol bark extract of *Yucca periculosa* (Asparagaceae) contained some active compounds against insect development. Of these active constituents, resveratrol had growth regulatory activity against *S. frugiperda* [68].

The current Gas Chromatography–Mass Spectrometry (GC–MS) analysis of *I. carnea* methanolic leaf crude extract showed the presence of 44 different bioactive compounds. The identified compounds were fatty acids, aliphatic hydrocarbons, polycyclic sesquiterpenes, phenolic compounds, steroids and alcohols. These constituents are known (Supplementary Materials—Table S1) for broad insecticidal and antimicrobial activities [29,30,31,32,33,34,35,36,37,38,39,40,41,42,43,44,45,46,47,48,49,50,51,52,53,54,55].

The present results are consistent with those of [69,70,71,72]. In 2023, [69] stated that the methanolic flower extract of *I. carnea* contains germacrene D (12.44%), n-hexadecanoic acid (12.15%), and caryophyllene (11.28%) with various biological activities: antimicrobial, cytotoxicity, antioxidant, insecticidal, insect repellant, anticancer and anti-inflammatory [69]. The phytochemical constituents of *I. carnea* aerial parts methanol extract were investigated by [70]. They stated that this extract has many bioactive secondary metabolites, with high levels of phenolics and flavonoids, and adequate amounts of alkaloids, saponins, glycosides and terpenes [70]. Using stem distillation, the essential oils (EO) of *I. carnea* arial parts were collected and their biological activity was identified and determined by [71]. They reported that this EO has antimicrobial activity against *E. coli*, *Klebsiella*, *Pseudomonas*, *Salmonella* and *Staphylococcus* spp. A total of 31 compounds (depending on GC–MS analysis) were identified, mainly terpenes (94.82). These compounds are grouped in six classes as follows: oxygenated monoterpenes, sesquiterpene hydrocarbons, oxygenated sesquiterpenes, diterpene hydrocarbons, carotenoid and apocarotenoid-derived compounds [71]. The chemical profile of *I. tricolor* and *I. fistulosa* leaves were characterized by GC–MS which led to the identification of 22 and 33 compounds, respectively [72]. The petroleum ether extracts of both species elicited antioxidant, anticholinesterase and anti-inflammatory activities. The extracts contained several fatty acids (saturated and unsaturated), aliphatic and phenylated hydrocarbons and oxygenated sesqueterpenses [72].

These chemical constituents and bioassay data collectively justify *I. carnea* methanolic leaf extract as a novel botanical control option against *S. frugiperda*. There is a dire need for further investigations of *I. carnea* methanolic leaf extract under field conditions and their effects on the non-target organisms.

### 4.2. Effect of I. carnea Methanolic Leaf Extract on Some Biochemical Components of S. frugiperda Larvae

The current biochemical effects of *I. carnea* methanolic extract on *S. frugiperda* larvae may be attributed to the presence of diverse secondary metabolites with insecticidal activities, as mentioned above. These bioactive constituents detected in the current study are known to interfere with insect metabolism and disrupt enzyme activity, leading to mortality and growth inhibition. These findings support the current observations of significant reductions in enzymatic activities of AChE and other esterases, while the peroxidase activity was markedly elevated in treated *S. frugiperda* larvae compared to the control. The effect of the plant extract on insect enzyme activity is one of the most important biochemical parameters that affect the physiology of the insect body. These effects may be increased or decreased the enzyme activity.

Peroxidase plays an important role in qualifying oxidative stress by regulating reactive oxygen species (ROS) generated under toxic exposure. A marked increase in peroxidase activity was observed in the current study. Therefore, monitoring the activity of enzymes provides insight into the physiological and biochemical impacts of the methanolic extract of *I. carnea* against *S. frugiperda* larvae. The decreased activity of enzyme may be due to the denaturation or destroyed of enzyme. This pattern may suggest a neurophysiological disruption and activation of oxidative stress in the treated *S. frugiperda* larvae leading to paralysis and death. Similar findings were reported by [73], they examined the toxicity of alkaloid fraction of *Nicotiana glauca* (Solinaceae) against the third instar larvae of FAW. The results showed significant inhibition of AST, ALT, ACP, AchE and GST enzyme activity, while the activity of ALP was elevated in the treated larvae compared with the control [73]. A concentration of 20 µg/mL of *I. tricolor* petroleum ether leaf extract caused a significant decrease in the AchE activity [72].

The total protein, lipid and carbohydrate content of the *S. frugiperda* larvae treated with the methanolic leaf extract of *I. carnea* are decreased in the present study. The reduction in total protein may be due to their degradation and the possible use of protein for metabolism. The present results are consistent with that of [63]. They reported that the total protein level in the whole body tissue of *C. quinquefasciatus* larvae treated with apigenin (extracted from *Jatropha gossypifolia*) decreased to 92% of control at 24 h post treatment [63].

## 5. Conclusions

In conclusion, the methanolic leaf extract of *Ipomoea carnea* exhibited strong concentration-and time-dependent larvicidal activity against *Spodoptera frugiperda*, under laboratory conditions. The biochemical findings confirmed that *I. carnea* methanol leaf extract disrupts the enzymatic functions of the treated *S. frugiperda* larvae. The treatment significantly decreased the esterase’s activity, while it increased the peroxidase activity. The reduction of the antioxidant activity of esterases and the high mortality achieved even at moderate concentrations highlights the potential of *I. carnea* methanolic leaf extract as novel promising insecticide. There is a dire need for further research to validate the field efficacy of *I. carnea* methanolic leaf extract and to assess its potential effects on non-target organisms before its practical application in integrated pest management programs.

## Figures and Tables

**Table 1:** Corrected larval mortality percentage of *Spodoptera frugiperda* treated as second larval instar with different concentrations of *Ipomoea carnea* methanolic leaf extract.

Con. (%)	Mean Corrected Larval Mortality Percentage (±S.E.) After Time of Treatment					
	6 h	12 h	24 h	36 h	48 h	72 h
1%	30.00 ± 5.00 d	36.67 ± 3.33 c	40.74 ± 9.26 b	59.63 ± 10.37 b	59.63 ± 10.37 c	64.81 ± 10.19 c
2%	41.67 ± 1.67 c	43.33 ± 3.33 c	48.15 ± 1.85 b	58.52 ± 1.48 b	60.37 ± 0.37 c	67.04 ± 2.96 c
3%	56.67 ± 3.33 b	61.67 ± 3.33 b	65.19 ± 4.81 ab	67.04 ± 2.96 b	75.56 ± 4.44 bc	77.41 ± 2.59 bc
4%	58.33 ± 1.67 b	68.33 ± 3.33 ab	87.04 ± 12.96 a	88.89 ± 11.11 a	92.59 ± 7.41 ab	92.59 ± 7.41 ab
5%	71.67 ± 3.33 a	78.33 ± 3.33 a	88.89 ± 11.11 a	92.59 ± 7.41 a	96.30 ± 3.70 a	96.30 ± 3.70 a
F value	65.08	47.96	16.50	22.40	37.48	39.05
LSD	10.06	12.044	25.279	21.673	17.612	17.24
Prob.	0.0001	0.0001	0.0001	0.0001	0.0001	0.0001

Conc. = concentration; LSD = least significant difference; Prob. = probability. The same letter means no statistically significant differences, while different letters show statistically significant differences.

**Table 2:** Corrected larval mortality percentage of *S. frugiperda* treated as fourth larval instar with different concentrations of *I. carnea* methanolic leaf extract.

Con. (%)	Mean Corrected Larval Mortality Percentage ± S.E. After Time of Treatment					
	6 h	12 h	24 h	36 h	48 h	72 h
1%	25.00 ± 10.00 a	40.00 ± 0.00 c	41.67 ± 1.67 d	48.33 ± 1.67 d	51.67 ± 1.67 c	74.56 ± 0.44 b
2%	25.00 ± 10.00 a	46.67 ± 3.33 bc	55.00 ± 0.00 c	60.00 ± 0.00 c	66.67 ± 1.67 b	79.47 ± 5.53 ab
3%	28.33 ± 11.67 a	48.33 ± 1.67 b	60.00 ± 2.89 bc	65.00 ± 0.00 b	66.67 ± 1.67 b	82.81 ± 7.19 ab
4%	35.00 ± 15.00 a	58.33 ± 1.67 a	65.00 ± 0.00 b	66.67 ± 1.67 b	73.33 ± 1.67 b	92.98 ± 7.02 a
5%	35.00 ± 15.00 a	61.67 ± 3.33 a	73.33 ± 1.67 a	76.67 ± 1.67 a	93.33 ± 6.67 a	92.98 ± 7.02 a
F value	1.28	92	226.17	370.20	96.56	37.68
LSD	35.27	6.954	5.136	4.193	9.376	17.05
Prob.	0.34	0.0001	0.0001	0.0001	0.0001	0.0001

Conc. = concentration; LSD = least significant difference; Prob. = probability. The same letter means no statistically significant differences, while different letters show statistically significant differences.

**Table 3:** Toxicological effect of *I. carnea* leaf methanolic extract at 48 h post-treatment on *S. frugiperda* larvae.

Stage	LC_50_	LC_90_	LC_95_	LC_99_	Slope	Slope +/−	*Chi* Square	*p*	r
Second instar	0.968	4.453	6.863	15.4499	1.933	0.336	12.1968	0.0067	0.8821
Fourth instar	1.052	8.221	14.726	43.9458	1.435	0.313	7.8878	0.0484	0.8342

*p* = probability; r = correlation coefficient.

**Table 4:** Latent effect of *I. carnea* methanolic leaf extract (LC_50_) against 2nd larval instar of *S. frugiperda*.

Stage	* Mean Weight (g) ± SE		Percentage of Mean Reduction	*t*-Test Value	*p*-Value
	Control	Treated			
Sixth instar larvae	0.07 ± 0.00	0.03 ± 0.00	60.78%	1.22 × 10^−15^	*** (<0.0001)
Pupal stage	0.18 ± 0.01	0.08 ± 0.01	58.11%	7.09 × 10^−11^	** (<0.001)

* = Mean weight of 60 larvae. *t*-test was used to compare treated groups with control; ** = significant; *** = highly significant. Weight reduction (%) = [(Mean weight of control − Mean weight of treated)/Mean weight of control] × 100 [20].

**Table 5:** HPLC-quantified data of secondary bioactive compounds presents in the crude methanolic leaf extract of *I. carnea* and GC–MS analysis of the crude methanolic leaf extract of *I. carnea*, showing separation of many compounds according to their retention times *.

Integration Results							
No.	Peak Name	Retention Time min	Area mAU*min	Height mAU	Relative Area%	Relative Height %	Amount ug/mL
1	Anthranilic acid	1.173	0.041	0.220	3.30	3.11	6.4254
2	Ellagic acid	1.627	0.031	0.382	2.51	5.39	4.8875
3	Naringin acid	1.825	0.074	0.807	5.97	11.41	11.6469
4	Salicylic acid	4.145	0.032	0.163	2.58	2.30	5.0305
5	Transferrulic acid	4.782	0.005	0.142	0.41	2.01	0.8047
6	Resveratrol	5.562	0.812	1.757	65.76	24.83	128.1935
7	Apigenin	8.225	0.036	0.050	2.91	0.70	5.6693
Total:			1.031	3.519	83.44	49.76	
RT **	Compound Name			Area%	Molecular Formula	Molecular Weight	Cas#
4.02	Hexanoic acid, 2-methyl-			0.02	C_7_H_14_O_2_	130	4536-23-6
4.72	Trans-2-Pentenoic acid			0.31	C_5_H_8_O_2_	100	13991-37-2
5.07	9-Oxabicyclo [3.3.1]nonan-2-one, 5-hydroxy-			0.1	C_8_H_12_O_3_	156	35519-67-6
5.32	4-Oxocyclopentane-1,2-dicarboxylic acid			0.03	C_7_H_8_O_5_	172	NA
5.41	9-Oxabicyclo [3.3.1]nonan-2-one, 5-hydroxy-			0.05	C_8_H_12_O_3_	156	35519-67-6
5.69	1-Methoxy-3-hydroxymethylheptane			0.02	C_9_H_20_O_2_	160	71052-95-4
6.7	2-Decen-1-ol, (E)-			0.01	C_10_H_20_O	156	18409-18-2
6.79	Cyclohexanone, 2-(hydroxymethyl)-			0.01	C_7_H_12_O_2_	128	5331-08-8
7.25	4H-Pyran-4-One, 3-Hydroxy-2-Methyl-			0.07	C_6_H_6_O_3_	126	118-71-8
7.33	Pyrimidine-4,6-diol, 5-methyl-			0.4	C_5_H_6_N_2_O_2_	126	18337-63-8
8.21	2,3-Dimethylfumaric acid			0.03	C6H8O4	144	NA
14.27	Eicosane, 2-cyclohexyl-			1.48	C26H52	364	4443-56-5
14.84	1-Tetradecanol			0.03	C_14_H_30_O	214	112-72-1
14.92	1-Dodecanol, 3,7,11-trimethyl-			0.01	C_15_H_32_O	228	6750-34-1
15.01	1-Hexadecanol, 2-methyl-			0.01	C_17_H_36_O	256	2490-48-4
15.07	Dodecanoic acid, 3-hydroxy-			0.02	C_12_H_24_O_3_	216	1883-13-2
15.31	Dodecanoic acid, 2-hexen-1-yl ester			0.21	C_18_H_34_O_2_	282	NA
15.47	á-copaene			0.28	C15H24	204	NA
15.76	1,12-Tridecadiene			0.55	C_13_H_24_	180	21964-48-7
16.01	6-Nonenal, 3,7-dimethyl-			0.03	C_11_H_20_O	168	NA
16.38	Caryophyllene oxide			0.02	C_15_H_24_O	220	1139-30-6
18.46	10-Methyl-E-11-tridecen-1-ol propionate			0.12	C_17_H_32_O_2_	268	NA
18.51	9,12-Octadecadienoic acid (Z,Z)-			0.18	C_18_H_32_O_2_	280	60-33-3
18.82	Oleic Acid			0.12	C_18_H_34_O_2_	282	112-80-1
20.87	1-Nitro-á-d-arabinofuranose, tetraacetate			0.03	C_13_H_17_NO_11_	363	NA
21.06	14-Pentadecynoic acid, Methyl Ester			0.15	C_16_H_28_O_2_	252	56909-04-7
23.63	Neophytadiene			1.14	C_20_H_38_	278	504-96-1
24.99	7-Hexadecenoic acid, methyl ester, (Z)-			0.24	C_17_H_32_O_2_	268	56875-67-3
25.17	Hexadecanoic acid, methyl ester			8.85	C_17_H_34_O_2_	270	112-39-0
26.25	n-Hexadecanoic acid			0.65	C_16_H_32_O_2_	256	57-10-3
26.81	9,12,15-Octadecatrienoic acid, 2,3-bis(acetyloxy)propyl ester, (Z,Z,Z)-			0.17	C_25_H_40_O_6_	436	55320-02-0
28.35	9,12,15-Octadecatrienoic acid, methyl ester, (Z,Z,Z)-			59.8	C_19_H_32_O_2_	292	301-00-8
28.7	Phytol			13.57	C_20_H_40_O	296	150-86-7
28.9	Methyl stearate			3.14	C_19_H_38_O_2_	298	112-61-8
29.29	9,12,15-Octadecatrienoic acid, (Z,Z,Z)-			2.53	C_18_H_30_O_2_	278	463-40-1
29.8	9,12,15-Octadecatrienoic acid, methyl ester, (Z,Z,Z)-			0.41	C_19_H_32_O_2_	292	301-00-8
37.67	Methyl 8,11,14,17-eicosatetraenoate			0.33	C_21_H_34_O_2_	318	NA
37.72	Methyl 8,10-octadecadiynoate			0.78	C_19_H_30_O_2_	290	NA
38.45	1,25-Dihydroxyvitamin D3, TMS derivative			0.4	C_30_H_52_O_3_Si	488	55759-94-9
40.14	9,12,15-Octadecatrienoic acid, 2,3-bis(acetyloxy)propyl ester, (Z,Z,Z)-			0.1	C_25_H_40_O_6_	436	55320-02-0
41.27	5,8,11-Eicosatrienoic acid, (Z)-, TMS derivative			0.16	C_23_H_42_O_2_Si	378	NA
41.49	Cholest-5-En-3-Ol (3á)-			0.2	C_27_H_46_O	386	57-88-5
41.93	Cholestan-3-ol, 2-Methylene-, (3á,5à)-			0.09	C_28_H_48_O	400	22599-96-8
42.51	(+)-à-Tocopherol			2.37	C_29_H_50_O_2_	430	59-02-9

* The biological activity (indicated with references) of the identified compounds is shown in the Supplementary Materials—Table S1 [29,30,31,32,33,34,35,36,37,38,39,40,41,42,43,44,45,46,47,48,49,50,51,52,53,54,55]. RT ** = retention time, Cas# = registration number in Chemical Abstracts Service, NA = Not Available.

**Table 6:** Effect of *I. carnea* methanolic leaf extract on certain biochemical aspects of *S. frugiperda* larvae treated as fourth larval instar.

Treatment	Total Carbohydrate Content (mg/g b. wt.)	Total Protein Content (mg/g b.wt.)	Total Lipid Content (mg/g b.wt.)	Acetylcholin-Esterase (nmol/min/mg Protein)	α-Esterase (nmol/min/mg Protein)	β-Esterase (nmol/min/mg Protein)	Peroxidase (nmol/min/mg Protein)
Methanol (Control)	168.33 ± 2.03	403.67 ± 2.19	319.33 ± 1.2	233.33 ± 1.86	68.33 ± 2.03	47.33 ± 1.45	0.24 ± 0.01
*Ipomoea carnea* extract	150.67 ± 0.88	343.67 ± 1.45	287.00 ± 1.15	162.33 ± 3.28	51.00 ± 0.58	30.67 ± 1.86	0.50 ± 0.01
t value	7.99 **	22.86 **	19.40 **	18.83 **	8.22 **	7.07 **	15.30 **
Prob > |t|	0.0013	0.0001	0.0001	0.0001	0.0012	0.0021	0.0001

** = significant b. wt. = body weight nmol/min/mg = nanomol/minute/milligram protein.

## Data Availability

The original contributions presented in this study are included in the article. Further inquiries can be directed at the corresponding authors.

## Supplementary Materials

The following supporting information can be downloaded at: https://www.mdpi.com/article/10.3390/insects17090888/s1.
