# Supplementary material for: Larvicidal and Biochemical Effects of Ipomoea carnea (Convolvulaceae) Methanolic Leaf Extract Against Spodoptera frugiperda (Lepidoptera: Noctuidae) Under Laboratory Conditions
Source: Insects. 2026 Aug 25;17(9):888. doi: 10.3390/insects17090888 (PMC13607087; doi:10.3390/insects17090888)
Supplement: Supplementary file 1 [file insects-17-00888-s001.zip › insects-4420434-Supplementary Table S1.pdf]

Final-Supplementary Table S1

Chemical classification	Isolated Compounds	Biological activity	references
medium-chain fatty acids, carboxylic acids, and aliphatic organic compounds.	Hexanoic acid, 2-methyl-	Antimicrobial activity; insecticidal / repellent effects; allelopathic / plant interaction; metabolic role; cytotoxic / bioactive potential.	[29,30]
An unsaturated monocarboxylic acid	trans-2-Pentenoic acid	Antimicrobial activity; insecticidal / antifeedant effects; plant defense and allelopathy; metabolic and biochemical role; cytotoxic and bioactive effects	[31,32,33].
bicyclic organic molecule, specifically belonging to the oxabicyclo, ketone, **and** alcohol (hydroxy) families.	9 Oxabicyclo [3.3.1] nonan-2-one, 5-hydroxy-	Antimicrobial activity; insecticidal and antifeedant effects; growth-regulating and developmental effects; cytotoxic and bioactive properties; plant defense role	[33,34,35].
a dicarboxylic acid, a cyclopentanone	4-Oxocyclopentane-1,2-dicarboxylic acid	Antimicrobial activity; chelating and enzyme interference; insecticidal / growth effects; allelopathic and plant-related effects; cytotoxic and metabolic effects	[36,37,38]
bicyclic organic compound, specifically a bridged lactol/ether derivative featuring both ketone and alcohol functional groups	9-Oxabicyclo [3.3.1]nonan-2-one, 5-hydroxy-	Antimicrobial activity; insecticidal and antifeedant effects; insect growth regulation; cytotoxic and reactive properties; role in plant defense	[34,35,36]
**an** ether **and an** aliphatic alcohol	1-Methoxy-3-hydroxymethylheptane	Antimicrobial activity; insecticidal / repellent effects; volatile / semiochemical role; synergistic effects; cytotoxic / metabolic Effects	[33,39,40].
fatty alcohols, aliphatic unsaturated alcohols	2-Decen-1-ol, (E)-	Antimicrobial activity; insecticidal and repellent effects; semiochemical role (chemical signaling); cytotoxic and membrane effects; synergistic effects in plant extracts	[33,39,40].
**a** cyclic ketone **and a** primary alcohol,	Cyclohexanone, 2-(hydroxymethyl)-	Antimicrobial activity; insecticidal / behavioral effects; cytotoxic and biochemical effects; role in plant defense; synergistic contribution	[33,39,40].
**a** pyranone, **an** organoheterocyclic compound, **and a** gamma-pyrone derivative.	4h-pyran-4-one, 3-hydroxy-2-methyl-	Antioxidant activity; antimicrobial activity; metal chelating ability; insecticidal / growth effects; cytotoxic and protective dual role	[33,39,40]
Nitrogen compound	Pyrimidine-4,6-diol, 5-methyl-	Antimicrobial activity; insecticidal / growth regulatory effects; cytotoxic and enzyme inhibition; synergistic role	[33,34,39]

Final-Supplementary Table S1

Fatty acid	2,3-Dimethylfumaric acid	Antimicrobial activity; insecticidal / antifeedant effects; cytotoxic / reactive properties; plant Defense / allelopathic Role	[31,32,33]
Cyclic hydrocarbon	Eicosane, 2-cyclohexyl-	Antimicrobial activity; insecticidal / antifeedant effects; hydrophobic / synergistic role; cytotoxic / biochemical Effects	[33,35,39].
Alcohol	1-Tetradecanol	Antimicrobial activity; insecticidal / antifeedant effects; hydrophobic / supportive role(lipophilic); cytotoxic / biochemical effects	[31,32,33]
Alcohol	1-Dodecanol, 3,7,11-trimethyl-	Antimicrobial activity; insecticidal / antifeedant effects; hydrophobic / supportive role; cytotoxic / biochemical effects	[31,32,33].
Alcohol	1-Hexadecanol, 2-methyl-	Antimicrobial activity; insecticidal / antifeedant effects; hydrophobic / synergistic role; cytotoxic / biochemical effects	[31,32,33]
Fatty acid	Dodecanoic acid, 3-hydroxy-	Antifeedant behavior; growth inhibition; larval and pupal deformities	[31,32,33].
Alkene	á-copaene	Antimicrobial activity: insecticidal / semiochemical effects (can alter feeding or oviposition behavior); cytotoxic / biochemical effects; synergistic role	[33,35,41]
Alkene	1,12-Tridecadiene	Antimicrobial activity; insecticidal / antifeedant effects (likely acts as a sociochemical); hydrophobic / synergistic role; cytotoxic / biochemical effects	[33,35,41]
Aldehyde	6-Nonenal, 3,7-dimethyl-	Antimicrobial activity; insecticidal / antifeedant effects; cytotoxic / biochemical effects; synergistic role	[33,35,41]
Polycyclic Sesquiterpenes	Caryophyllene oxide	Antimicrobial activity; insecticidal / antifeedant effects; cytotoxic / biochemical effects; synergistic role	[33,35,41]
Ester	10-Methyl-E-11-tridecen-1-ol propionate	Antimicrobial activity; insecticidal / antifeedant effects; hydrophobic / synergistic role; cytotoxic / biochemical effects	[33,35,41]

Final-Supplementary Table S1

Fatty acid	9,12-Octadecadienoic acid (Z, Z)-	Antimicrobial activity; insecticidal / antifeedant effects; cytotoxic / biochemical effects ; synergistic role	[31,32,42].
Fatty acid	Oleic Acid	Antimicrobial activity; insecticidal / antifeedant effects; cytotoxic / biochemical effects; synergistic role	[31,32,42]
Ester	1-Nitro-á-d-arabinofuranose, tetraacetate	Antimicrobial activity; insecticidal / antifeedant effects; cytotoxic / biochemical effects; synergistic role	[43,44,45]
Ester	14-Pentadecynoic acid, methyl ester	Antimicrobial activity; insecticidal / antifeedant effects; cytotoxic / biochemical effects; synergistic role	[31,32,46]
alkene	Neophytadiene	Antimicrobial activity; insecticidal / antifeedant effects; cytotoxic / biochemical effects; synergistic role	[33,35,41].
Ester	7-Hexadecenoic acid, methyl ester, (Z)-	Antimicrobial activity; insecticidal / antifeedant effects; cytotoxic / biochemical effects; synergistic role	[31,32,64].
Ester	Hexadecanoic acid, methyl ester	Antimicrobial activity; insecticidal / antifeedant effects; cytotoxic / biochemical effects; synergistic role	[31,32,46]
Fatty acid	n-Hexadecanoic acid	Antimicrobial activity; insecticidal / antifeedant effects; cytotoxic / biochemical effects; synergistic role	[31,32,42]
Ester	9,12,15-Octadecatrienoic acid, 2,3-bis(acetyloxy)propyl ester, (Z,Z,Z)	Antimicrobial activity; insecticidal / antifeedant effects; cytotoxic / biochemical effects; synergistic role	[31,32,42]
Ester	9,12,15-Octadecatrienoic acid, methyl ester, (Z,Z,Z)-	Antimicrobial activity; insecticidal / antifeedant effects; cytotoxic / biochemical effects; synergistic role	[31,32,46]
Alcohol	Phytol	Antimicrobial activity; insecticidal / antifeedant effects; cytotoxic / biochemical effects; synergistic role	[33,35,47]
Ester	Methyl stearate	Antimicrobial activity; insecticidal / antifeedant effects; cytotoxic / biochemical effects; synergistic role	[31,32,46]

Final-Supplementary Table S1

Fatty acids	9,12,15-Octadecatrienoic acid, (Z,Z,Z)-	Antimicrobial activity; insecticidal / antifeedant effects; cytotoxic / biochemical effects; synergistic role	[31,42,46]
ester	9,12,15-Octadecatrienoic acid, methyl ester, (Z, Z,Z)-	Antimicrobial activity; insecticidal / antifeedant effects; cytotoxic / biochemical effects; synergistic role	[31,32,46]
Ester	Methyl 8,11,14,17-eicosatetraenoate	Antimicrobial activity; insecticidal / antifeedant effects; cytotoxic / biochemical effects; synergistic role	[31,32,46]
Ester	Methyl 8,10-octadecadiynoate	Antimicrobial activity; cytotoxic / anticancer potential; anti-inflammatory effects; enzyme modulation; potential insecticidal / antifeedant activity	[31,32,46]
vitamin	1,25-Dihydroxyvitamin D3, TMS derivative	Hormonal / growth-regulatory effects; antifeedant / cytotoxic effects; synergistic role	[48,49,50]
Ester	9,12,15-Octadecatrienoic acid, 2,3-bis(acetyloxy)propyl ester, (Z,Z,Z)-	Antimicrobial activity; insecticidal / antifeedant effects; cytotoxic / biochemical effects; synergistic role	[31,32,46]
Fatty acid	5,8,11-Eicosatrienoic acid, (Z)-, TMS derivative	Antimicrobial activity; insecticidal / antifeedant effects; cytotoxic / biochemical effects; synergistic role	[31,32,46]
Steroid	Cholest-5-en-3-ol (3á)-	Membrane / structural role; insecticidal / antifeedant effects; cytotoxic / biochemical effects; synergistic role	[48,51,52]
Steroid	Cholestan-3-ol, 2-methylene-, (3á,5à)-	Membrane / structural role; insecticidal / antifeedant effects; cytotoxic / biochemical effects; synergistic role	[31,51,52]
Vitamin	(+)-à-Tocopherol	Antioxidant / protective effects; insecticidal / antifeedant effects; cytotoxic / biochemical effects; synergistic role	[53,54,55]
